# Feasibility of Elemental and Microstructural Differentiation of Land Snail Eggs from *Bradybaena ravida* and *Cathaica fasciola*

**DOI:** 10.3390/biology15090721

**Published:** 2026-05-02

**Authors:** Yiya Wang, Fengjiang Li, Siyi Peng, Jiujiang Zhao, Linghao Zhao, Yajie Dong, Dongyang Sun, Naiqin Wu

**Affiliations:** 1 National Research Center for Geoanalysis, Beijing 100037, China; yiyawang@163.com (Y.W.);; 2 State Key Laboratory of Lithospheric and Environmental Coevolution, Institute of Geology and Geophysics, Chinese Academy of Sciences, Beijing 100029, China; 18874081765@163.com (S.P.);; 3 College of Earth and Planetary Sciences, University of Chinese Academy of Sciences, Beijing 100049, China

**Keywords:** eggs, mollusca, *Gastropoda*, chemical elements, microstructure, ecology

## Abstract

Although species identification is crucial for land-snail eggs, limited effort has been made to identify the species responsible for producing the eggs. In this study, we used laser ablation inductively coupled plasma mass spectrometry (LA–ICP–MS) to measure 54 elements in both the eggshells and adult shells of *Bradybaena ravida* and *Cathaica fasciola* and we used scanning electron microscope (SEM) to analyze the microstructure of the eggshells of the two species. Our results reveal that while the concentrations of Sr, Na, Mg, P, and Ba in the adult shells of the two species are not distinct, they are distinct or partially distinct between their eggshells, indicating that these elements have the potential to differentiate the eggs of the two species. Moreover, the eggshells of *C. fasciola* exhibit a blocky morphology without cavities, whereas those of *B. ravida*, while also blocky, contain irregular cavities. These distinct elemental and microstructural characteristics enable the effective differentiation of the eggs of *B. ravida* and *C. fasciola*. Our study demonstrates the feasibility of a critical microscopic methodology for identifying land-snail eggs at the genus/species level, thereby facilitating deeper exploration of their value in understanding biological, climatic, and ecological changes.

## Simple Summary

Eggs serve as unique materials for reconstructing biological, climatic, and ecological changes. Although species identification of eggs is essential for such studies, it has received insufficient attention. Land snails are one of the most abundant egg-laying invertebrates. However, the eggs of *Bradybaena ravida* and *Cathaica fasciola* are similar in size and shape and thus cannot be readily distinguished by the naked eye. Here we report the first attempt to identify eggs of these two species using microscopic characteristics. Our results show that the concentrations of Sr, Na, Mg, P, and Ba in the eggshells of *B. ravida* and *C. fasciola* differ either completely or partially. For example, the Sr content ranges from 429 to 510 µg/g in *B. ravida* eggshells, but from 197 to 248 µg/g in *C. fasciola* eggshells. Microstructurally, *C. fasciola* eggshells display a blocky structure without cavities, whereas *B. ravida* eggshells contain irregular cavities. This study suggests that species-level discrimination of land-snail eggs based on microscopic features is feasible. Our work provides a starting point for more systematically designed investigations. Future studies should extend to eggs from more snail species and explore additional diagnostic traits to differentiate land-snail eggs at the genus or species level.

## 1. Introduction

Eggs hold particular value in studies of biology, climatology, and ecology for the following reasons [1]. First, from a biological perspective, oviparity is the most prevalent mode of reproduction because more than 90% of total animal species, including both vertebrates and invertebrates, are oviparous [2]. Second, from a climatological standpoint, egg hatching can be strongly impacted by abrupt climatic cooling events occurring during the reproductive season [3]. Eggs require specific temperature ranges for successful hatching; for example, the hatching temperatures of the snails investigated are mostly between 15 and 25 °C [3,4,5]. When seasonal abrupt climatic events cause temperatures to fall outside these thermal ranges, numerous eggs fail to hatch [3]. Third, from an ecological perspective, eggs are closely linked to population and community dynamics, as hatching success or failure directly influences the abundance of the populations or communities involved [6].

Until now, the eggs extensively investigated in geological records have been mainly of dinosaurs and birds [7]. Although the biological value of these vertebrate eggs is significant, their stratigraphic distribution is often discontinuous, which severely limits their use in climatology and ecology [1,7]. In contrast, oviparous invertebrates can lay numerous eggs that are likely to form continuous and abundant distributions in geological archives [1,2]. Among invertebrates, arthropods and molluscs are the most prolific egg-laying groups; however, arthropod eggs are generally unlikely to be abundantly preserved as fossils due to their uncalcified eggshells [8]. Fortunately, the calcified eggshells of terrestrial molluscs can be abundantly and well preserved in geological archives, particularly in carbonate-rich deposits such as loess [1,9,10].

Well-preserved intact eggs in loess deposits indicate that they failed to hatch [9,10]. Hatching failure can result from abrupt cooling events during the reproductive season, as temperature is the most critical factor influencing the hatching success of land-snail eggs. Seasonal abrupt cooling events can drive temperatures beyond the optimal hatching range [1,3,9,10], a relationship that has been validated by surface soil investigations of land-snail eggs [3]. Based on these findings, land-snail eggs have been used to reconstruct the history of seasonal cooling events over the past five glacial–interglacial cycles [9,10]. However, in most cases, the inability to differentiate land-snail eggs at the species level limits a deeper understanding of the climatic changes recorded by these egg fossils.

Identification of land-snail eggs at the genus or species level is crucial. Biologically, it has been shown that the characteristics of different plant species are often recorded in reproductive organs such as pollen and seeds [11,12]. Similarly, eggs—one of the main reproductive stages in animals—also have the potential to document identifiable characteristics at the species or genus level through the microstructure, elemental composition, shape, and size of the eggshells, or a combination of these traits [5,13]. For example, land snail species of different sizes produce eggs of different sizes: most of the 2–3-mm land snails produce eggs of 0.6–0.8 mm in diameter, and 10-mm snails lay eggs of approximately 1.2 mm in diameter [5,13]. However, land-snail eggs cannot be accurately assigned to species or genus based solely on egg size. Earlier research in the 1970s revealed that the microstructure of eggshells varies among different land snail species [14]; regrettably, however, land-snail egg fossils were not extensively found or reported in the 1970s. Consequently, the climatic value of eggs was not exploited, and species- or genus-level identification of land-snail eggs remained scarce [1]. In 2016, however, scientists from Poland published results on the eggshell microstructure of *Vallonia costata* and *V. pulchella*, clearly demonstrating that the eggs of these two species exhibit distinct microstructural differences [15]. This work indicates that, in some cases, land-snail eggs can be identified to species level using the microstructure of their eggshells.

**Figure 1:**
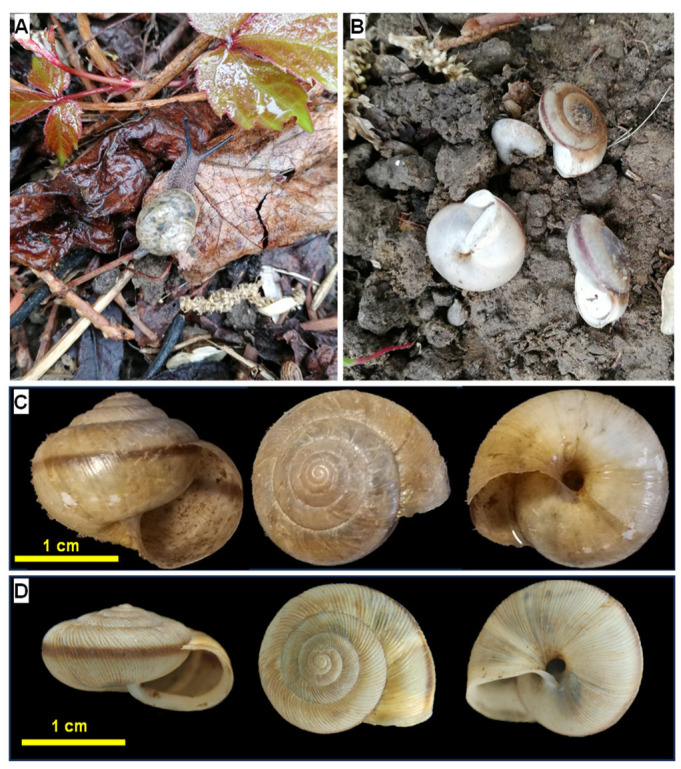
Field photographs of *Bradybaena ravida* and *Cathaica fasciola* in Beijing (April 2022). (**A**) An active individual of *B. ravida*. (**B**) Several dormant individuals of *C. fasciola*. (**C**) Lateral, apical, and umbilical views of *B. ravida*. (**D**) Lateral, apical, and umbilical views of *C. fasciola*.

As indigenous species, *Bradybaena ravida* (currently accepted as *Acusta ravida*) and *Cathaica fasciola* (Figure 1) are two of the most widespread land snail species in mainland China, making it feasible to obtain sufficient individuals for laboratory cultivation [16]. They, or their respective genera, were also identified in Neogene aeolian deposits of the Chinese Loess Plateau in 2004 and 2006 [17,18,19,20]. Unfortunately, however, no information is currently available on the fossil eggs of *C. fasciola* and *B. ravida* because fossil land-snail eggs were not studied until 2019 [1]. In addition to their indigenous status, *B. ravida* and *C. fasciola* were selected for this study because of their relatively large body size (exceeding 15 mm in maximum dimension), which makes them more manageable for cultivation.

**Figure 2:**
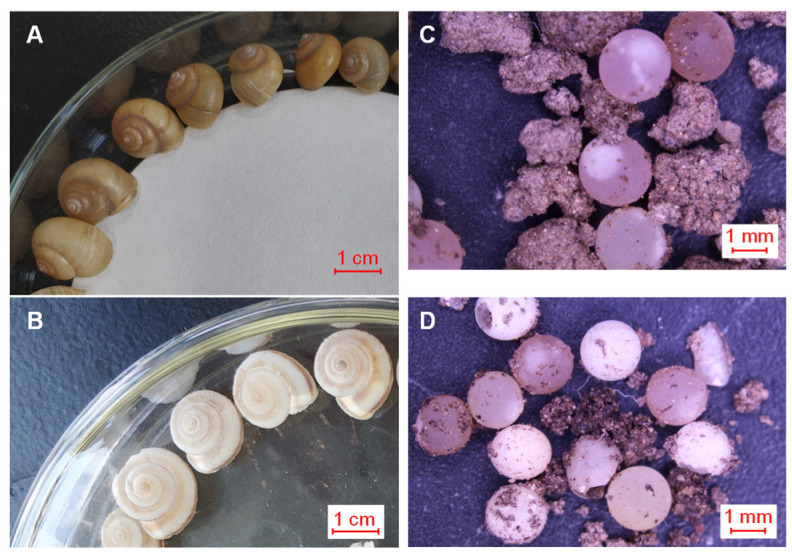
Photographs of *Bradybaena ravida* and *Cathaica fasciola* and their eggs. (**A**,**B**) show *B. ravida* and *C. fasciola*, respectively, with distinctly different shell shapes and sizes visible to the naked eye. (**C**,**D**) show the eggs of *B. ravida* and *C. fasciola*, respectively, which exhibit a similar shape and size to the naked eye.

Although *B. ravida* and *C. fasciola* differ greatly in their shell shapes and sizes (Figure 1C,D), their eggs are similar in shape and size (Figure 2), making identifiable characteristics invisible to the naked eye. In this study, we investigate several features that are not visible to the naked eye, including the elemental composition and microstructure of the eggshells of *B. ravida* and *C. fasciola*. The objective is to determine these microscopic characteristics and demonstrate the feasibility of a microscopic methodology for identifying land-snail eggs.

## 2. Materials and Methods

Our field and laboratory work were conducted in Beijing (39°54′ N, 116°23′ E), which lies in the East Asian monsoon region [21]. The present-day climate of Beijing is characterized by a pronounced seasonality driven by the alternation of the East Asian winter and summer monsoons. In winter, the East Asian winter monsoon prevails, bringing dry and cold conditions. The January average temperature of −4.6 °C results in almost all land snails remaining inactive [16]. In spring, the average temperature rises to 13.1 °C, which enables most land snail species to become active after hibernation and to reproduce [21]. In summer, the East Asian summer monsoon transports warm, moist air masses into the Beijing area, resulting in heavy rainfall and warm conditions. The July average temperature of 25.8 °C for Beijing is optimal for the growth and development of land snails [16]. In autumn, temperatures decrease, with an average of approximately 12.4 °C [21], approaching the minimum temperature required for land snail activity [3,5]. By late autumn, conditions gradually become unfavorable for the growth and development of land snails, and with the onset of winter, land snails in Beijing enter hibernation [16].

Identifying the species that laid the eggs is the first step in determining the identifiable characteristics of the eggs of different land snail species. In April 2022, adult individuals of *B. ravida* and *C. fasciola*, either active or dormant (with epiphragms sealing their apertures), were collected from similar habitats (bare or sparsely covered by low-density leaf litter) at different locations (Figure 1). A total of 28 individuals of *B. ravida* were collected from the campus of the Institute of Geology and Geophysics, Chinese Academy of Sciences, Chaoyang District, Beijing, China. Thirty-five individuals of *C. fasciola* were collected from the yard of No. 14, Fuxingmenwai Street, Xicheng District, Beijing. Individuals of each species were brought to our laboratory at the National Research Center for Geoanalysis, Beijing, where they were separately reared and fed the same diet. Under favorable conditions (temperature of 19–26 °C, relative humidity of 60–80%, sufficient food, and natural photoperiod conditions in the laboratory) maintained in an artificial climate incubator, they laid eggs after approximately two to four weeks. Eggs were collected and stored separately in a refrigerator to ensure the correspondence between the two species and their eggs.

Six eggshells each of *B. ravida* and *C. fasciola* were analyzed for both elemental composition and microscopic morphology, and six adult shells of each species were used for elemental analysis. We measured 54 elements in the adult shells of *B. ravida* and *C. fasciola* and in their eggshells using laser ablation inductively coupled plasma mass spectrometry (LA–ICP–MS) at the Key Laboratory of In Situ Elemental Microprobe and Speciation Analysis, China Geological Survey. Land snail shell samples were taken from near the lip of six adult shells of each species. The samples were cleaned in an ultrasonic bath, and their surfaces were further cleaned with anhydrous ethanol and ultrapure water to remove possible contamination. Given the fragility of the eggshells during the washing process, six clean eggshells of each species from a single parent were selected. The samples were mounted on glass slides and directly analyzed for elemental contents using LA–ICP–MS. The instrumentation consisted of an NWR 193UC laser ablation system coupled with a Thermo Finnigan Element II sector field inductively coupled plasma mass spectrometer [22,23].

The snail shells and eggshells were ablated using a spot size of 40 µm, a repetition rate of 8 Hz, and a laser energy density of approximately 5 J/cm^2^. Helium was used as the carrier gas for the aerosol generated by laser ablation. The silicate reference material NIST 610 was used to calibrate element concentrations, and the carbonate reference material MACS-3 was analyzed as a monitoring sample. Data processing was performed using Iolite software (version 4.0). For carbonate measurements, Ca was used as the internal standard [24] and was excluded from the reported results. Since the main component of snail shells and eggs is CaCO_3_, the internal standard element Ca content was taken as 40% to calculate the concentrations of other elements. Using this method to determine the elemental composition of MACS-3, the precision and accuracy of the analyses were better than 10% for most elements compared to the reference values.

Using R software (version 4.5.0) [25] (code archived in the Supplementary Information), principal component analysis (PCA), non-metric multidimensional scaling (nMDS), and canonical variate analysis (CVA) were performed on the elemental composition of land-snail eggshells of *B. ravida* and *C. fasciola*. PCA and nMDS were used to assess the compositional separation between the two egg groups. As a distance-based method that requires fewer distributional assumptions than PCA’s orthogonal transformation, nMDS yielded more distinct group differentiation. CVA maximizes inter-group differentiation, quantifies the relative contributions of individual elements to group separation, and generates an equation for predicting the identity of unknown specimens. However, due to the limited sample size in the current dataset, a reliable predictive equation could not be derived; robust formulation will require expanded sampling in future studies. Additionally, after application of the Shapiro–Wilk normality test on the concentrations of five elements with identifiable characteristics in the two egg groups, an independent-samples *t*-test was performed using R software (version 4.5.0) [25].

Micro-area morphology and back scattered electron (BSE) images of the samples were obtained at the National Research Center for Geoanalysis using a Zeiss Sigma 500 field emission scanning electron microscope (SEM) equipped with a Bruker QUANTAX XFlash6|30 X-ray spectrometer energy-dispersive spectroscopy (EDS) system (Carl Zeiss AG, Oberkochen, Germany). Six cleaned eggshells from each species of *B. ravida* and *C. fasciola* were used for SEM sample preparation. The SEM was operated under high vacuum mode, with working voltages of 1–15 kV, working distances (WD) of 3–10 mm, and an objective aperture of 60 µm.

## 3. Results

The eggs of *B. ravida* and *C. fasciola* are spherical and white, with sizes ranging mainly from 1.5 to 2 mm (Figure 2; Figure S1). Thus, the eggs of the two species cannot reliably be distinguished by size or shape alone. We therefore sought to identify differences between the eggs of the two species by analyzing their elemental composition and microstructure.

### 3.1. Elemental Abundance in Eggshells and Adult Shells of B. ravida and C. fasciola

We measured 54 elements in the adult shells and eggshells of *B. ravida* and *C. fasciola* (Table 1). As shown in Table 1, 42 elements (from Al to Lu) were excluded from further consideration because they exhibited either very low concentrations (maximum < 10 µg/g) or at least five concentration values of zero, indicating limited potential as identifiable features. Therefore, these elements are unlikely to be useful for distinguishing the eggs of the two species. In contrast, 12 elements (Si, Na, Mg, K, Fe, Sr, P, Ba, B, Cr, As, and Mn—in descending order of maximum concentration) are relatively abundant, with maximum concentrations exceeding 10 µg/g and, at most, two concentration values of zero across all shell and eggshell samples of the two species (Table 1, Table S1).

**Figure 3:**
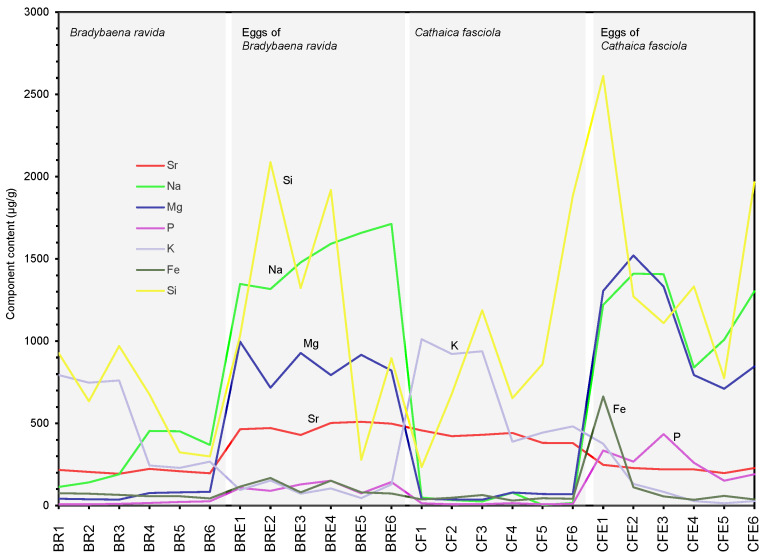
Concentrations (µg/g) of Si, Na, Mg, K, Fe, Sr, and P in the adult shells and eggshells of *Bradybaena ravida* and *Cathaica fasciola*. BR1–BR6: sample numbers for *B. ravida* adult shells; BRE1–BRE6: sample numbers for *B. ravida* eggshells. CF1–CF6: sample numbers for *C. fasciola* adult shells; CFE1–CFE6: sample numbers for *C. fasciola* eggshells.

Among the elements measured, Si is the most abundant, particularly in the eggshells of the two species (Figure 3). In the six eggshells of *B. ravida*, Si content ranges from 278 to 2087 µg/g, with four eggshells exceeding 1000 µg/g. The Si content range in *B. ravida* eggshells is wider than that in the adult shells of *B. ravida* (299–970 µg/g) (Table 1). Similarly, the six eggshells of *C. fasciola* have Si contents ranging from 775 to 2612 µg/g, with two eggshells having values higher than those in the adult shells of *C. fasciola*. Across the two species, the Si content decreases with the progression of the life cycle from egg to adult (Figure 3).

As shown in Table 1 and Figure 3, the Na content of the adult shells of *B. ravida* ranges from 114 to 454 µg/g, whereas in the eggshells of *B. ravida* it ranges from 1317 to 1713 µg/g, indicating that the adult shells have a substantially lower Na content than the eggshells. Similarly, the Na content in the adult shells of *C. fasciola* ranges from 4.37 to 79 µg/g—markedly lower than that in its eggshells (840–1410 µg/g). Although the Na content in the eggshells of the two species shows some overlap, four eggshells of *B. ravida* have a Na content exceeding 1450 µg/g, whereas all eggshells of *C. fasciola* have a Na content below 1450 µg/g (Table 1).

For the eggshells of both species, the Mg content ranges from 716 to 996 µg/g in the eggshells of *B. ravida* and from 710 to 1520 µg/g in the eggshells of *C. fasciola*. Some eggshells of *C. fasciola* have an Mg content exceeding 1000 µg/g, whereas all the eggshells of *B. ravida* have an Mg content lower than 1000 µg/g. The Mg contents of the eggshells of the two species overlap at values below 1000 µg/g (Table 1, Figure 3).

The K content of the adult shells of *B. ravida* ranges from 244 to 793 µg/g, which is approximately 200–600 µg/g higher than that of its eggshells (45.5–152 µg/g) (Table 1, Figure 3). Similarly, the K content in all individuals of *C. fasciola* (388–1011 µg/g) is substantially higher than that of its eggshells (14.3–376 µg/g) (Table 1, Figure 3). Among the 12 eggs of the two species, 10 have overlapping K values, indicating that the K content of the eggshells of the two species is similar.

The Fe content of the adult shells of the two species and their eggshells is low, with most values not exceeding 100 µg/g. In the adult shells of *B. ravida*, the Fe content ranges from 43.5 to 75.2 µg/g, which is slightly lower than that of its eggshells (73.4–167 µg/g) (Table 1, Figure 3). In *C. fasciola*, the Fe content ranges from 30.7 to 64 µg/g in the adult shells and from 35.5 to 663 µg/g in its eggshells. Notably, the Fe content in *B. ravida* eggshells overlaps with that in *C. fasciola* eggshells, and the Fe content range in *C. fasciola* eggshells is relatively wide.

The Sr content of the adult shells of the two species and their eggshells ranges from 193 to 510 µg/g (Table 1). The Sr content of the eggshells of *B. ravida* ranges from 429 to 510 µg/g, whereas for the eggshells of *C. fasciola* it ranges from 197 to 248 µg/g (Table 1, Figure 3), indicating that the Sr contents of the eggshells of the two species comprise non-overlapping ranges. Similarly, the Sr content of the adult shells of the two species also shows no overlap (Figure 3).

Compared to the elements described above, the P content is relatively low in both the adult shells of the two species and in their eggshells, with the highest value not exceeding 335 µg/g. In *B. ravida*, the adult shells have a P content ranging from 8.06 to 26.8 µg/g, while the eggshells have P contents between 74.5 and 152 µg/g (Table 1, Figure 3). There is little overlap in the P content between the two egg types. All six eggshells of *C. fasciola* have a P content of at least 152 µg/g, whereas the five eggshells of *B. ravida* fall below 152 µg/g, with only one eggshell reaching 152 µg/g (Table 1).

**Figure 4:**
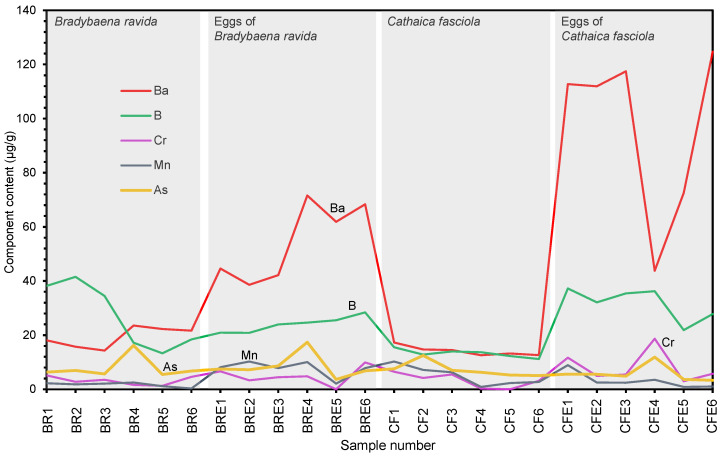
Concentrations (µg/g) of Ba, B, Cr, As, and Mn in the adult shells and eggshells of *Bradybaena ravida* and *Cathaica fasciola*. BR1–BR6: adult shells of *B. ravida*; BRE1–BRE6: eggshells of *B. ravida*. CF1–CF6: adult shells of *C. fasciola*; CFE1–CFE6: eggshells of *C. fasciola*.

Among the elements not mentioned above, Ba, B, Cr, As, and Mn show relatively high concentrations (Table 1, Figure 4). In *B. ravida*, the Ba content ranges from 14.3 to 23.5 µg/g in the adult shells and from 38.6 to 71.6 µg/g in the eggshells (Table 1, Figure 4), indicating that the adult shells have a lower Ba content than the eggshells. In *C. fasciola*, the Ba content ranges from 12.6 to 17.3 µg/g in the adult shells and from 43.8 to 125 µg/g in the eggshells (Table 1, Figure 4). All the eggshells of *B. ravida* have a Ba content below 80 µg/g, whereas only two eggshells of *C. fasciola* fall below this threshold.

The B content of the adult shells of both snail species and their eggshells does not exceed 41.5 µg/g (Table 1, Figure 4). In *B. ravida*, the B content ranges from 13.3 to 41.5 µg/g in the adult shells and from 20.9 to 28.4 µg/g in the eggshells; in *C. fasciola*, the B content ranges from 11.2 to 15.5 µg/g in the adult shells and from 21.8 to 37.2 µg/g in the eggshells (Table 1). Thus, the B content of the eggshells of *B. ravida* and *C. fasciola* overlaps within the range of 21.8 to 28.4 µg/g. Values above 28.4 µg/g are found exclusively in the eggshells of *C. fasciola*, whereas values below 21.8 µg/g occur only in the eggshells of *B. ravida*.

As shown in Table 1 and Figure 4, the Cr content of the adult shells of *B. ravida* (1.25–5.12 µg/g) falls entirely within the range of that of its eggshells (0.00–9.95 µg/g). In *C. fasciola*, the Cr content of the adult shells (0.00–6.47 µg/g) largely overlaps with that of its eggshells (2.98–18.7 µg/g), and the values are relatively low and indistinguishable. Considering only the eggshells of the two species, the Cr values of *B. ravida* eggs show no substantial difference from those of *C. fasciola* eggs.

The adult shells of *B. ravida* and *C. fasciola* and their eggs have a similar As content (Figure 4). The As content ranges from 5.45 to 16.2 µg/g in the adult shells of *B. ravida* and from 5.08 to 12.5 µg/g in those of *C. fasciola* (Table 1, Figure 4). Their eggshells also show comparable As values: 3.64–17.4 µg/g for *B. ravida* eggs and 3.31–11.8 µg/g for *C. fasciola* eggs (Table 1).

The Mn content is very low (no higher than 10.2 µg/g) and similar in both the adult shells of *B. ravida* and *C. fasciola* and their eggs. The Mn content of the adult shells of *B. ravida* ranges from 0.28 to 2.47 µg/g, which largely overlaps with that of its eggshells (2.08–10.2 µg/g) (Table 1, Figure 4). Five eggshells of *B. ravida* have a higher Mn content than the adult snails. *C. fasciola* and its eggs show similar Mn content ranges (0.87–10.2 µg/g and 0.89–8.90 µg/g, respectively) (Table 1), with most values overlapping (Figure 4). Therefore, the Mn content does not differ substantially between the two species or between their eggs.

**Figure 5:**
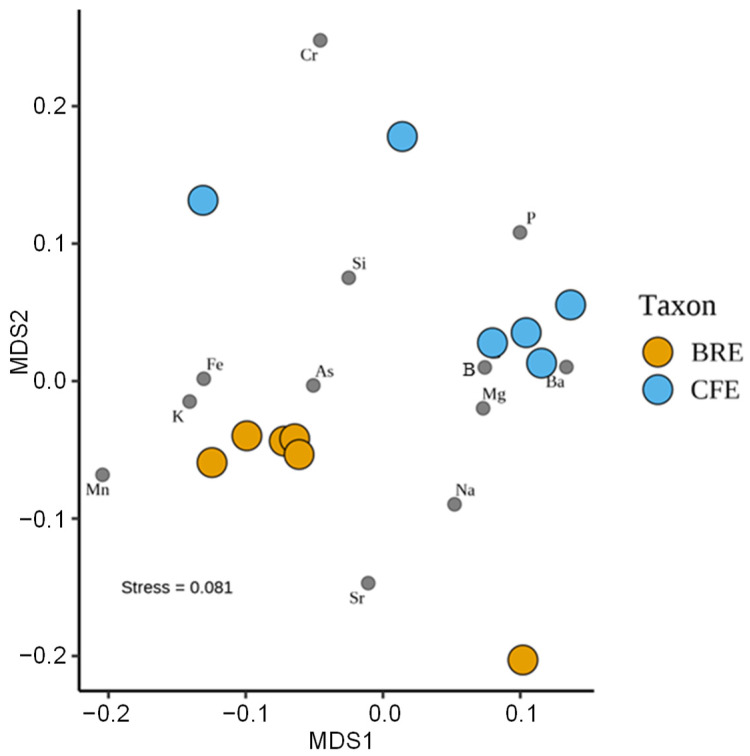
Results of non-metric multidimensional scaling (nMDS) analysis of the elemental composition (Si, Na, Mg, K, Fe, Sr, P, Ba, B, Cr, As, and Mn) of the eggshells of the land snails *Bradybaena ravida* and *Cathaica fasciola*. BRE: *B. ravida* eggshells. CFE: *C. fasciola* eggshells.

*T*-test results show that the concentrations of Ba, Na, P, and Sr have statistically significant differences between the two egg groups (*p* < 0.05), among which P shows a highly significant difference (*p* < 0.01), Sr shows an extremely significant difference (*p* < 0.001), while Mg shows no statistically significant difference (*p* > 0.05) (Table S2). The PCA results show that the elemental compositions of the two egg groups are well separated, indicating that the eggs of the two species differ in elemental composition (Figure S2). The nMDS results reveal a clearer separation between the two egg groups (Figure 5). The CVA results show that the eggs of *B. ravida* tend to have higher Sr and Na values, accompanied by moderate Mn and As values, whereas the eggs of *C. fasciola* tend to have higher P, Ba, B, and Mg values, associated with lower Cr, Si, Fe, and K values (Figure S3). Overall, the results of these three methods are largely consistent, further confirming that the two egg groups differ in elemental composition.

### 3.2. Microstructural Characteristics of B. ravida and C. fasciola Eggshells

**Figure 6:**
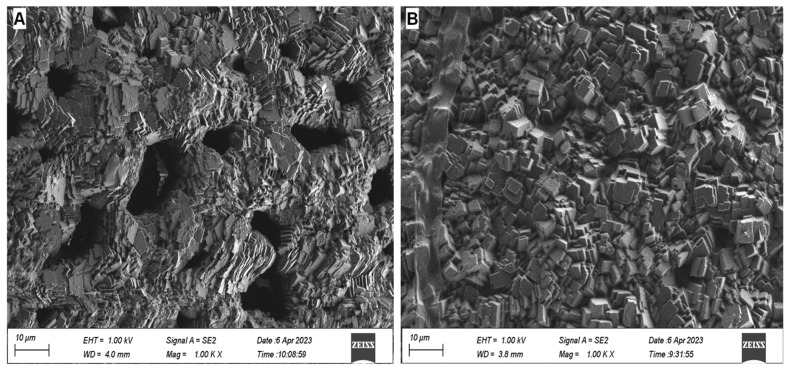
Microstructural morphology of land-snail eggshells. (**A**) *Bradybaena ravida* eggs showing a blocky texture with irregular cavities. (**B**) *Cathaica fasciola* eggs showing a blocky morphology without cavities.

The microstructures of the eggshells of *B. ravida* and *C. fasciola* differ markedly, although both are composed of calcite crystals (Figure 6). SEM observations reveal that the calcite crystals in *B. ravida* eggshells are twinned, thin, and sheet-like, exhibiting layered/plate-like stacks with visible laminae and step-like growth traces. Most notably, numerous cavities of varying sizes and shapes are present within the eggshell microstructure of *B. ravida* (Figure 6A). These cavities are predominantly elongated and irregular, with the largest dimensions typically below 20 µm. In contrast, the eggshell microstructure of *C. fasciola* consists of relatively large, twinned crystals with smooth, flat crystal faces, showing clearly distinguishable three sets of perfect cleavage, drusy aggregates, rhombohedral crystal form, and cleavage features. Most distinctly, cavities are absent in the microstructure of *C. fasciola* eggshells (Figure 6B).

## 4. Discussion

In the 1970s, differentiation of land-snail eggs at the species level was noted, and the microstructures of the eggs from several snail species were found to differ [14]. More recently, Polish malacologists discovered that the eggs of *Vallonia costata* and *V. pulchella* exhibit distinctly different microstructures [15]. Additionally, egg size can sometimes be used to broadly constrain eggs to a few genera or species, but it cannot definitively assign them to a specific species [3,5,13]. Elemental analysis of land-snail eggs has not been extensively conducted and thus has not previously been used for genus or species identification [13,14,15]. In summary, whether microscopic characteristics can reliably be used to differentiate land-snail eggs at the genus or species level requires further investigation. Our investigation of the elemental composition and microstructure of *B. ravida* and *C. fasciola* and their eggs indicates that certain elements and/or microstructural features can potentially differentiate the eggs of the two species. These potentially identifiable characteristics are summarized in Table 2. They indicate the feasibility of using microscopic characteristics to separate land-snail eggs to genera or species.

The differences in elemental composition between the adult shells of the two species are very limited (Figure 3 and Figure 4). In contrast, the eggs of the two species exhibit complete or partial differences in the concentrations of several elements, primarily Sr, Na, Mg, P, and Ba. This suggests that although the adult shells of the two species differ greatly in shape, their elemental compositions cannot be used for species identification; however, the elemental compositions of the eggs can effectively distinguish between the two species, with certain elemental characteristics being unique to the eggs of each species, thereby providing a basis for using composition to separate eggs at the species level.

The Sr content in the eggs of *B. ravida* and *C. fasciola* shows no overlap (Figure 3). As noted above, the Sr content ranges of the two species differ markedly: 429–510 µg/g for *B. ravida* eggs and 197–248 µg/g for *C. fasciola* eggs (Table 1). Therefore, the Sr content can be used to fully differentiate between the eggs of *B. ravida* and *C. fasciola*.

The Na content can partially differentiate the eggs of *B. ravida* and *C. fasciola*. Using a threshold of 1450 µg/g, eggs with a Na content exceeding this value belong exclusively to *B. ravida*, whereas eggs with a Na content below 1450 µg/g are more likely to be from *C. fasciola* and less likely from *B. ravida* (Table 1 and Table 2, Figure 3).

Mg is also useful for distinguishing between the eggs of *B. ravida* and *C. fasciola*. Eggs with a Mg content exceeding 1000 µg/g can be definitively assigned to *C. fasciola*, whereas those with Mg content below 1000 µg/g cannot be unambiguously identified. In the latter case, eggs are more likely to belong to *B. ravida* and less likely to *C. fasciola* (Table 1 and Table 2; Figure 3).

The P content of the eggs of *B. ravida* ranges from 74.5 to 152 µg/g, whereas the eggs of *C. fasciola* have a minimum P content of 152 µg/g (Table 1, Figure 3). If the P content of an egg exceeds 152 µg/g (i.e., not including 152 µg/g), it can definitively be assigned to *C. fasciola* (Table 1). Conversely, if the P content is significantly lower than 152 µg/g, the egg can be assigned to *B. ravida*. Using 150 µg/g as a threshold, eggs with a P content above 150 µg/g are most likely from *C. fasciola*, whereas those with a P content below 150 µg/g are most likely from *B. ravida*.

All eggs of *B. ravida* have a Ba content lower than 80 µg/g, whereas four of the six eggs of *C. fasciola* have a Ba content exceeding 80 µg/g (Table 1). Therefore, among the eggs of the two species examined, those with a Ba content greater than 80 µg/g can be unequivocally assigned to *C. fasciola*. In contrast, eggs with a Ba content below 80 µg/g could belong to either species, although they are more likely to be from *B. ravida* than from *C. fasciola* (Table 1 and Table 2; Figure 4). The B content can serve as an identification marker to differentiate the two egg types only when its value exceeds 28.4 µg/g, as values above this threshold are found exclusively in the eggs of *C. fasciola* (Table 1, Figure 4). However, the B content is very low among all elements with the potential for distinguishing the eggs of the two species (Table 1), and therefore the most accurate measurements are required.

It is worth noting that species-level identification of an egg can be more accurate when multiple characteristic elements are used simultaneously. If an egg exhibits several elemental features that consistently point to either *B. ravida* or *C. fasciola*, it can reliably be identified. For example, consider an egg of unknown species suspected to belong to one of these two species. If the egg shows an Sr content exceeding 400 µg/g, Na content above 1450 µg/g, and P content below 150 µg/g, it can unequivocally be assigned to *B. ravida*. Conversely, if the egg has an Mg content below 1000 µg/g, P content lower than 150 µg/g, and Ba content under 80 µg/g, it is highly likely to originate from *B. ravida*.

Elements that cannot be used to differentiate the eggs of the two species include K, Fe, Si, Cr, Mn, As, B, as well as Al, Ti, Zn, Li, and the 42 trace elements listed in Table 1. These elements exhibit either highly similar or overlapping concentrations in the eggs of *B. ravida* and *C. fasciola* or occur at very low (even negligible) levels (Table 1).

Compared with the characteristic elements in the eggs of *B. ravida* and *C. fasciola*, egg microstructure is clearly more useful for assigning eggs to species (Figure 6). Indeed, the utility of egg microstructure in differentiating land-snail eggs at the family, genus, and even species level has long been recognized [14]. For example, as summarized by Tompa (1976) [14], the ultrastructure of the eggs of *Euglandina guttata*, *E. rosea*, and *E. texasiana* is distinctly different and identifiable; the eggs of *Anguispira alternata* are also readily distinguishable from those of *A. kochi*; and the eggs of *Discus patula* can be differentiated from those of *D. cronkhitei* (currently known as *D. whitneyi*). More recently, Kuźnik-Kowalska and Proćków (2016) [15] discovered that SEM imaging of eggs of the minute land snails *Vallonia costata* and *V. pulchella* reveals clearly distinct microstructures, with the former characterized by larger calcite crystals and the latter by smaller ones. These studies indicate that eggs from the same genus can be definitively assigned to species.

Since *B. ravida* and *C. fasciola* belong to different genera, their egg microstructures should be even more readily distinguishable. The present study shows that the two species can be morphologically differentiated by eggshell microstructure: eggs of *B. ravida* exhibit a blocky texture with irregular cavities, whereas those of *C. fasciola* display a blocky morphology without such cavities (Figure 6). This striking microstructural difference may represent the most reliable characteristic for identifying the two species that laid the eggs. Our findings further support previous studies, suggesting that the eggs of each land-snail species possess a species-specific microstructure, pointing to genetic control as a likely—or primary—factor underlying the formation of eggshell microstructures [14,15], a hypothesis that warrants confirmation through further research.

The differences in elemental contents between the eggs of the two species directly indicate that *B. ravida* and *C. fasciola* differ in their ability to enrich certain characteristic elements in their eggs. However, the causes of this differential ability remain unclear. Two possible interpretations may be proposed. One interpretation relates to differences in food selection between the two land-snail species. *B. ravida* is polyphagous, feeding on a wide range of plants, whereas *C. fasciola* consumes a relatively narrower range of plants [16,26]. It is therefore possible that the higher Sr and Na contents in the eggs of *B. ravida* result from its broader diet. However, this explanation does not account for the lower Mg, P, and Ba contents observed in the eggs of *B. ravida*. Moreover, it is contradicted by the fact that in our experiment, both species were fed the same diet, suggesting that the influence of food on the elemental composition of the eggs is likely minor and not dominant. The other interpretation concerns genetic control over egg formation. Although no specific genetic studies are currently available for these two species, the fact that they belong to different genera supports the likelihood of substantial genetic divergence [13,16]. It is thus plausible that distinct genetic programs govern egg formation in the two species, leading to differences in certain characteristic elements. It is worth noting, however, that eggshells found in the wild or in the fossil record may exhibit greater variability in elemental composition due to the combined effects of both factors discussed.

In summary, our results are applicable for separating eggs of these two species and demonstrate feasibility for differentiating the eggs of other land snail species. If the elemental compositions and microstructural features identified here are unique to the eggs of *B. ravida* and *C. fasciola*, they can reliably be used to differentiate their eggs. If not, the present findings are still valuable for distinguishing eggs in assemblages dominated by these two species. The identified features of land-snail eggs would be useful for reconstructing climate histories recorded by fossil land-snail eggs in the 2.6 Ma loess deposits and, possibly, in the Miocene–Pliocene aeolian deposits of the Chinese Loess Plateau [17,18,19,20,27,28,29,30,31,32,33,34,35].

## 5. Conclusions

Despite its preliminary nature, this study presents the first microscopic characterization of *B. ravida* and *C. fasciola*—two widely distributed land snail species in mainland China—and their eggs. Although the two species differ markedly in adult shell shape and size, their eggs are similar in both size and shape, making visual identification by the naked eye unreliable. Our analyses of elemental concentrations and microstructures reveal that the eggs of *B. ravida* and *C. fasciola* exhibit complete or partial differences in the concentrations of Sr, Na, Mg, P, and Ba. Specifically, the Sr content ranges from 429 to 510 µg/g in *B. ravida* eggs and from 197 to 248 µg/g in *C. fasciola* eggs, indicating that Sr alone can fully differentiate the eggs of the two species. Eggs with a Na content exceeding 1450 µg/g belong to *B. ravida*, whereas those with a Na content below 1450 µg/g are more likely to be from *C. fasciola*. Eggs with an Mg content above 1000 µg/g are definitively from *C. fasciola*, while those with an Mg content below 1000 µg/g are more likely to be from *B. ravida*. Eggs with a P content below 150 µg/g are from *B. ravida*, whereas those with a P content above 150 µg/g are more likely from *C. fasciola*. Eggs with a Ba content exceeding 80 µg/g belong to *C. fasciola*, while those with a Ba content below 80 µg/g are more likely to be from *B. ravida*. Identification accuracy can be further improved by simultaneously considering multiple characteristic elements. In terms of microstructure, *C. fasciola* eggs exhibit a blocky morphology without cavities, whereas *B. ravida* eggs, though also blocky, contain irregular cavities—providing a clear microscopic feature for species discrimination. These results enable the differentiation of eggs of these two species and have potential for distinguishing others following further investigation.

Our study demonstrates that discriminating land-snail eggs at the species level based on elemental composition and microstructure is both feasible and promising. This study provides not only preliminary results but also a starting point for more systematically designed investigations. The approaches presented here offer a promising avenue for better utilizing land-snail eggs as a novel proxy for reconstructing biological, climatic, and ecological changes. Further work is needed to establish identifiable features in eggs of a broader range of species and individuals (including different parents) and to explore the causes of the observed marked differences in elemental and microstructural characteristics.

## Figures and Tables

**Table 1:** Elemental composition (µg/g) of adult shells and eggshells of *Bradybaena ravida* and *Cathaica fasciola*. Elements are listed in descending order of maximum concentration. Al, Ti, Zn, and Li are listed after Mn because they have five or more concentration values of zero. Sample numbers BR1–BR6 denote adult shells of *B. ravida*; BRE1–BRE6 denote eggshells of *B. ravida*. Similarly, CF1–CF6 denote adult shells of *C. fasciola*; CFE1–CFE6 denote eggshells of *C. fasciola*.

Content (µg/g)	Sample Number																							
	*Bradybaena ravida*						The Eggshells of *Bradybaena ravida*						*Cathaica fasciola*						The Eggshells of *Cathaica fasciola*					
	BR1	BR2	BR3	BR4	BR5	BR6	BRE1	BRE2	BRE3	BRE4	BRE5	BRE6	CF1	CF2	CF3	CF4	CF5	CF6	CFE1	CFE2	CFE3	CFE4	CFE5	CFE6
Si	927	634	970	676	324	299	1033	2087	1323	1919	278	896	234	680	1188	653	861	1883	2612	1272	1110	1332	775	1966
Na	114	142	190	454	452	369	1348	1317	1479	1592	1658	1713	48.4	34.0	25.0	79.0	4.37	15.7	1221	1410	1407	840	1009	1302
Mg	41.9	38.4	37.0	76.3	81.0	83.8	996	716	928	793	916	821	40.5	38.0	35.9	79.5	70.1	69.1	1305	1520	1332	793	710	846
K	793	747	761	244	229	267	94.7	152	72.6	105	45.5	129	1011	922	938	388	444	482	376	132	84.2	26.8	14.3	27.6
Fe	75.2	72.7	65.3	56.7	56.7	43.5	115	167	81.1	152	80.3	73.4	38.1	48.3	64.0	30.7	44.8	41.3	663	111	56.4	35.5	59.4	37.4
Sr	217	205	193	223	209	197	464	472	429	502	510	498	457	422	430	441	381	380	248	229	221	220	197	229
P	8.06	8.33	10.4	16.5	22.0	26.8	107	90.1	129	152	74.5	144	13.7	8.24	9.33	15.3	8.16	10.6	335	267	434	260	152	191
Ba	18.1	15.7	14.3	23.5	22.3	21.7	44.6	38.6	42.2	71.6	61.9	68.3	17.3	14.7	14.5	12.6	13.3	12.6	113	112	117	43.8	72.6	125
B	38.2	41.5	34.5	17.2	13.3	18.4	20.9	20.9	24.0	24.6	25.5	28.4	15.5	12.8	14.0	13.7	12.3	11.2	37.2	32.1	35.4	36.2	21.8	27.8
Cr	5.12	2.75	3.51	1.60	1.25	4.60	6.60	3.29	4.45	4.80	0.000	9.95	6.47	4.19	5.45	0.30	0.000	3.10	11.6	4.93	5.46	18.7	2.98	5.76
As	6.33	6.95	5.65	16.2	5.45	6.73	7.50	7.25	8.67	17.4	3.64	6.78	7.59	12.5	7.01	6.26	5.27	5.08	5.61	5.51	4.88	11.8	3.68	3.31
Mn	2.20	1.80	2.12	2.47	1.11	0.28	8.15	10.2	7.84	10.0	2.08	7.82	10.2	7.15	6.30	0.87	2.29	2.67	8.90	2.47	2.41	3.53	0.89	0.98
Al	0.000	1.14	1.01	5.62	3.07	0.000	131	147	58.0	172	1.53	55.1	0.000	0.051	0.000	0.000	0.000	0.000	1044	124	35.2	602	8.36	36.2
Ti	0.000	0.25	0.000	0.000	2.22	0.000	71.0	25.6	12.0	6.60	3.51	0.000	0.000	0.000	0.000	1.66	1.09	0.000	38.7	260	2.76	7.31	0.000	0.90
Zn	227	0.000	0.022	0.000	0.29	3.34	2.67	2.37	0.84	3.05	0.000	3.17	0.26	0.29	0.000	0.000	0.074	0.000	3.13	1.38	1.97	0.000	0.19	0.000
Li	90.0	0.000	0.000	0.000	0.000	0.000	0.000	0.000	0.000	0.000	0.000	0.000	0.000	0.20	0.000	0.000	0.000	0.000	1.81	0.000	0.64	1.66	0.000	0.000
Cu	0.000	0.900	0.000	0.000	0.000	0.000	1.95	0.57	3.31	2.20	0.037	3.60	0.011	0.48	0.000	0.51	0.22	0.15	2.28	1.11	1.18	2.12	0.000	0.33
Ni	0.000	0.89	0.000	3.22	0.50	0.000	0.43	0.000	0.39	2.22	0.000	1.95	0.000	0.000	0.25	0.000	0.000	0.000	1.08	0.000	0.000	0.000	1.21	0.000
Sn	0.000	0.000	0.12	0.000	0.092	0.000	0.007	0.11	0.59	0.071	0.031	0.23	0.000	0.000	0.25	0.000	0.000	0.000	0.48	0.18	0.057	0.000	1.53	0.11
Pb	0.022	0.040	0.003	0.009	0.055	0.045	0.59	0.40	0.37	0.29	1.14	0.15	0.000	0.045	0.000	0.000	0.009	0.003	1.23	0.59	0.66	0.37	0.53	0.22
La	0.031	0.027	0.029	0.013	0.024	0.020	0.060	0.063	0.033	0.042	0.035	0.059	0.027	0.017	0.046	0.016	0.037	0.037	1.16	0.053	0.060	0.000	0.12	0.008
U	0.000	0.000	0.000	0.001	0.000	0.000	0.016	0.004	0.000	0.034	0.000	0.11	0.000	0.17	0.002	0.000	0.002	0.000	0.12	0.035	0.048	0.006	1.13	0.029
V	0.060	0.040	0.010	0.012	0.014	0.003	0.31	0.38	0.24	0.73	0.13	1.06	0.000	0.000	0.003	0.000	0.000	0.039	1.02	0.24	0.19	0.22	0.28	0.34
Rb	0.066	0.057	0.13	0.058	0.039	0.009	0.44	0.61	0.000	0.16	0.13	0.047	0.000	0.16	0.000	0.000	0.000	0.000	1.06	0.10	0.006	0.000	0.097	0.000
Nd	0.000	0.000	0.028	0.006	0.011	0.000	0.056	0.044	0.014	0.056	0.026	0.000	0.011	0.000	0.034	0.022	0.000	0.016	1.03	0.048	0.000	0.062	0.000	0.000
Y	0.000	0.000	0.000	0.019	0.007	0.004	0.040	0.068	0.008	0.071	0.000	0.033	0.000	0.007	0.015	0.014	0.014	0.000	1.02	0.066	0.041	0.000	0.12	0.002
Ce	0.005	0.015	0.008	0.013	0.024	0.006	0.096	0.23	0.039	0.37	0.015	0.096	0.022	0.053	0.026	0.023	0.020	0.035	0.83	0.076	0.038	0.015	0.093	0.007
Cd	0.000	0.084	0.000	0.17	0.000	0.000	0.000	0.67	0.83	0.18	0.52	0.000	0.15	0.000	0.000	0.000	0.000	0.10	0.000	0.000	0.068	0.000	0.090	0.000
Ag	0.062	0.007	0.000	0.013	0.000	0.000	0.095	0.021	0.000	0.023	0.000	0.007	0.000	0.000	0.000	0.010	0.024	0.000	0.025	0.009	0.000	0.747	0.000	0.000
Zr	0.087	0.000	0.000	0.011	0.11	0.000	0.026	0.024	0.17	0.59	0.000	0.12	0.21	0.070	0.000	0.000	0.000	0.17	0.60	0.035	0.000	0.076	0.004	0.000
Be	0.020	0.000	0.20	0.000	0.000	0.000	0.000	0.20	0.35	0.000	0.10	0.000	0.12	0.000	0.48	0.000	0.000	0.000	0.000	0.16	0.16	0.000	0.000	0.26
Ge	0.23	0.000	0.000	0.000	0.13	0.000	0.027	0.28	0.25	0.43	0.023	0.23	0.094	0.14	0.010	0.000	0.19	0.000	0.065	0.093	0.000	0.14	0.20	0.000
Ga	0.12	0.048	0.000	0.024	0.077	0.038	0.024	0.000	0.000	0.040	0.025	0.038	0.000	0.000	0.000	0.029	0.000	0.020	0.28	0.050	0.028	0.079	0.000	0.008
Gd	0.011	0.009	0.011	0.000	0.000	0.015	0.006	0.021	0.000	0.025	0.084	0.000	0.008	0.000	0.000	0.000	0.006	0.037	0.28	0.011	0.000	0.000	0.024	0.000
Pr	0.003	0.000	0.002	0.004	0.000	0.002	0.023	0.007	0.000	0.026	0.000	0.014	0.000	0.000	0.000	0.003	0.000	0.000	0.26	0.003	0.000	0.007	0.018	0.013
Co	0.053	0.031	0.023	0.053	0.000	0.039	0.20	0.14	0.064	0.20	0.000	0.037	0.000	0.011	0.088	0.11	0.000	0.073	0.25	0.057	0.000	0.000	0.023	0.13
Sb	0.000	0.000	0.000	0.022	0.000	0.049	0.000	0.026	0.039	0.074	0.077	0.000	0.000	0.065	0.021	0.001	0.000	0.023	0.030	0.041	0.024	0.24	0.028	0.000
Sm	0.000	0.000	0.000	0.000	0.000	0.000	0.000	0.018	0.000	0.000	0.000	0.000	0.000	0.000	0.000	0.021	0.000	0.037	0.24	0.000	0.000	0.000	0.022	0.000
Dy	0.000	0.000	0.000	0.010	0.000	0.000	0.018	0.029	0.031	0.000	0.000	0.043	0.000	0.010	0.000	0.000	0.000	0.008	0.17	0.026	0.000	0.000	0.035	0.000
Sc	0.000	0.000	0.057	0.000	0.002	0.021	0.049	0.087	0.020	0.13	0.071	0.010	0.000	0.000	0.000	0.070	0.000	0.070	0.16	0.021	0.000	0.14	0.000	0.000
Nb	0.000	0.000	0.000	0.003	0.000	0.000	0.13	0.000	0.005	0.000	0.011	0.019	0.006	0.000	0.003	0.000	0.004	0.000	0.062	0.10	0.000	0.060	0.000	0.000
Th	0.000	0.000	0.000	0.000	0.000	0.000	0.023	0.024	0.014	0.028	0.000	0.000	0.000	0.000	0.000	0.000	0.000	0.000	0.12	0.023	0.000	0.000	0.034	0.000
Cs	0.046	0.020	0.016	0.024	0.000	0.000	0.000	0.000	0.011	0.001	0.025	0.000	0.000	0.000	0.000	0.000	0.000	0.050	0.11	0.000	0.000	0.000	0.076	0.000
Er	0.000	0.002	0.005	0.000	0.007	0.018	0.000	0.000	0.000	0.025	0.000	0.000	0.000	0.015	0.005	0.006	0.006	0.000	0.10	0.000	0.000	0.014	0.010	0.000
Eu	0.000	0.000	0.002	0.000	0.001	0.011	0.013	0.000	0.000	0.000	0.000	0.000	0.000	0.000	0.009	0.000	0.000	0.000	0.074	0.000	0.008	0.000	0.000	0.000
Bi	0.000	0.000	0.006	0.000	0.000	0.007	0.006	0.015	0.000	0.006	0.000	0.000	0.002	0.070	0.000	0.000	0.000	0.009	0.025	0.004	0.000	0.003	0.000	0.009
In	0.017	0.000	0.007	0.013	0.000	0.000	0.000	0.000	0.000	0.016	0.000	0.004	0.000	0.000	0.000	0.000	0.000	0.000	0.009	0.025	0.002	0.046	0.018	0.000
Ho	0.003	0.000	0.000	0.000	0.000	0.000	0.004	0.000	0.000	0.000	0.000	0.000	0.005	0.000	0.000	0.002	0.000	0.000	0.030	0.011	0.000	0.042	0.000	0.009
Tb	0.002	0.000	0.000	0.001	0.000	0.000	0.003	0.000	0.000	0.004	0.007	0.000	0.000	0.001	0.000	0.004	0.003	0.000	0.032	0.003	0.000	0.005	0.000	0.000
Hf	0.007	0.000	0.000	0.007	0.000	0.000	0.000	0.031	0.000	0.000	0.000	0.000	0.000	0.000	0.000	0.013	0.000	0.000	0.000	0.000	0.000	0.000	0.000	0.000
Yb	0.013	0.000	0.000	0.007	0.007	0.009	0.000	0.000	0.000	0.000	0.000	0.000	0.010	0.024	0.000	0.005	0.011	0.000	0.000	0.000	0.000	0.000	0.000	0.000
Ta	0.000	0.000	0.000	0.000	0.000	0.000	0.011	0.005	0.000	0.013	0.000	0.007	0.000	0.000	0.000	0.000	0.009	0.000	0.004	0.000	0.000	0.021	0.000	0.000
Tm	0.000	0.003	0.000	0.000	0.000	0.000	0.004	0.003	0.010	0.000	0.000	0.000	0.000	0.000	0.003	0.000	0.000	0.000	0.010	0.000	0.000	0.005	0.000	0.000
Lu	0.000	0.000	0.000	0.000	0.004	0.000	0.000	0.000	0.004	0.005	0.000	0.000	0.000	0.000	0.003	0.000	0.000	0.000	0.002	0.000	0.000	0.001	0.000	0.000

**Table 2:** Five potentially identifiable elements and their concentration ranges, characteristic concentrations, egg numbers with characteristic concentrations, and microstructure in *Bradybaena ravida* and *Cathaica fasciola* eggshells.

Element	Data Type	Eggs of *Bradybaena ravida*		Eggs of *Cathaica fasciola*	
Sr	Concentration range (µg/g)	429–510		197–248	
	Characteristic concentrations (µg/g)	>400		<250	
	Egg numbers with characteristic concentrations	6		6	
Na	Concentration range (µg/g)	1317–1713		840–1410	
	Characteristic concentrations (µg/g)	>1450	<1450	<1450	
	Egg numbers with characteristic concentrations	4	2	6	
Mg	Concentration range (µg/g)	716–996		710–1520	
	Characteristic concentrations (µg/g)	<1000		>1000	<1000
	Egg numbers with characteristic concentrations	6		3	3
P	Concentration range (µg/g)	74.5–152		152–434	
	Characteristic concentrations (µg/g)	<150	>150	>150	
	Egg numbers with characteristic concentrations	5	1	6	
Ba	Concentration range (µg/g)	38.6–71.6		43.8–125	
	Characteristic concentrations (µg/g)	<80		>80	<80
	Egg numbers with characteristic concentrations	6		4	2
Microstructure	/	Blocky with irregular cavities		Blocky without any cavity	

## Data Availability

The data obtained by this work can be found in the text.

## Supplementary Materials

The following supporting information can be downloaded at: https://www.mdpi.com/article/10.3390/biology15090721/s1, Figure S1: Egg sizes of land snails *Bradybaena ravida* (currently accepted as *Acusta ravida*) and *Cathaica fasciola*; Figure S2: Results of principal component analysis (PCA) of Si, Na, Mg, K, Fe, Sr, P, Ba, B, Cr, As, and Mn in the eggs of the land snails *Bradybaena ravida* and *Cathaica fasciola*; Figure S3: Results of canonical variate analysis (CVA) of Si, Na, Mg, K, Fe, Sr, P, Ba, B, Cr, As and Mn in the eggs of land snails *Bradybaena ravida* and *Cathaica fasciola*; Table S1: Mean, standard deviation (SD) and median of elemental concentrations in *Bradybaena ravida* and *Cathaica fasciola* and their eggs; Table S2: *T*-test results of five elements that have the potential to differentiate eggs of land snails *Bradybaena ravida* and *Cathaica fasciola*; R code.

## Abbreviations

The following abbreviations are used in this manuscript:

LA-ICP-MS	laser ablation inductively coupled plasma mass spectrometry
PCA	principal component analysis
nMDS	non-metric multidimensional scaling
CVA	canonical variate analysis
BSE	back scattered electron
SEM	scanning electron microscope
EDS	energy-dispersive spectroscopy
WD	working distance
