# Supplementary material for: Feasibility of Elemental and Microstructural Differentiation of Land Snail Eggs from Bradybaena ravida and Cathaica fasciola
Source: Biology (Basel). 2026 May 2;15(9):721. doi: 10.3390/biology15090721 (PMC13162794; doi:10.3390/biology15090721)
Supplement: Supplementary file 1 [file biology-15-00721-s001.zip › biology-4249113-supplementary.pdf]

Supplementary Information

Feasibility of elemental and microstructural differentiation of land snail eggs from *Bradybaena ravida* and *Cathaica fasciola*

Yiya Wang[1], Fengjiang Li[2,*], Siyi Peng[2,3], Jiujiang Zhao[1], Linghao Zhao[1], Yajie Dong[2], Dongyang Sun[1], Naiqin Wu[2]

[1]National Research Center for Geoanalysis, Beijing 100037, China.

[2]State Key Laboratory of Lithospheric and Environmental Coevolution, Institute of Geology and Geophysics, Chinese Academy of Sciences, Beijing 100029, China.

[3]College of Earth and Planetary Sciences, University of Chinese Academy of Sciences, Beijing 100049, China.

[*]Corresponding author: Fengjiang Li (Email: fengjiangli@mail.iggcas.ac.cn).

This file includes:

Figures S1 to S3, Table S1, Table S2, and R code.

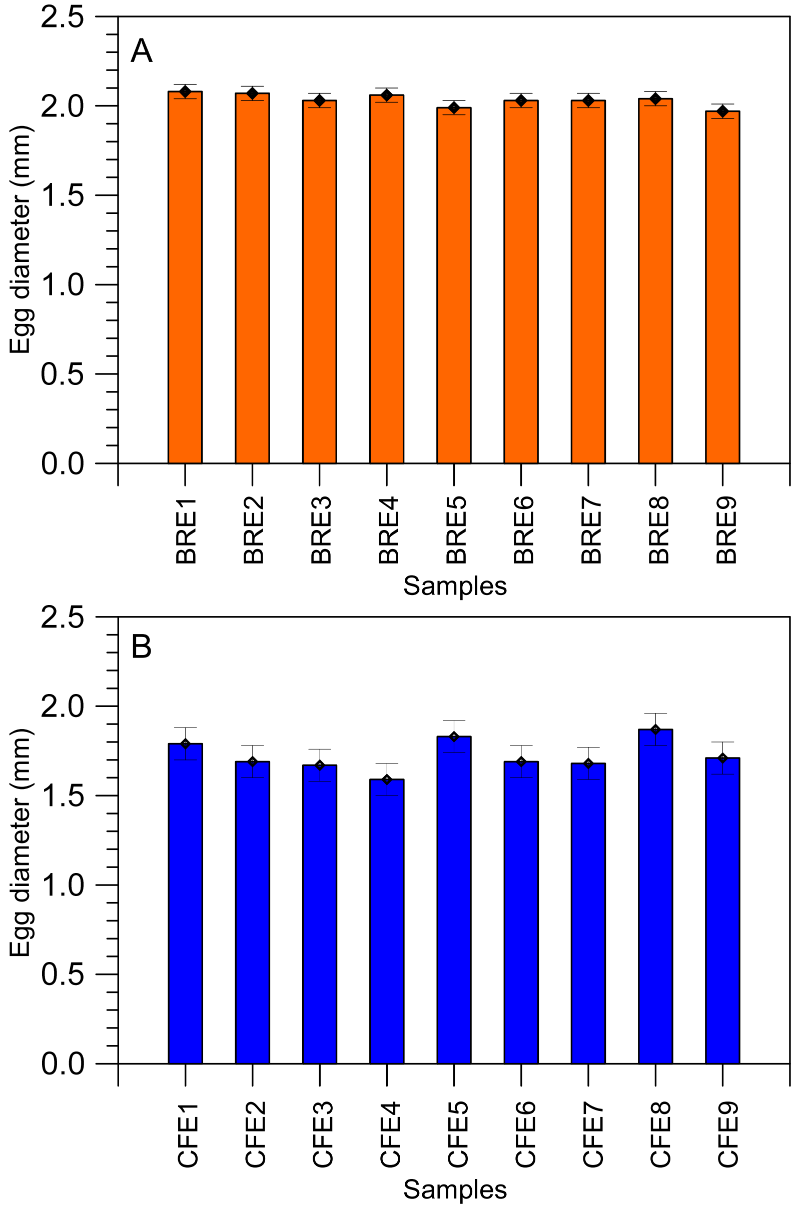

Figure S1. Egg sizes of land snails *Bradybaena ravida* (currently accepted as *Acusta ravida*) and *Cathaica fasciola*. A. Size of *Bradybaena ravida* eggs. B. Size of *Cathaica fasciola* eggs.

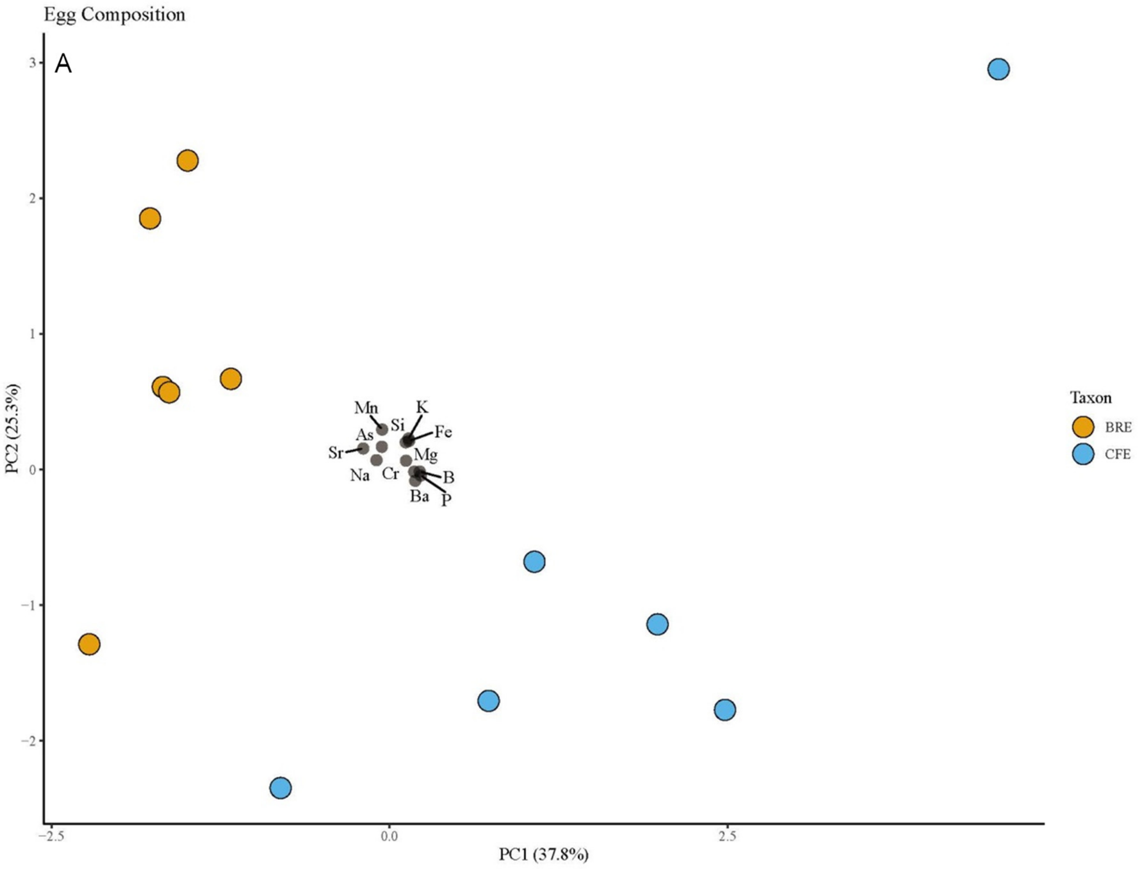

Egg Composition
A
PC2 (25.3%)
PC1 (37.8%)
3
2
1
0
−1
−2
−2.5
0.0
2.5
Mn
K
Si
Fe
As
Sr
Mg
Na
Cr
B
Ba
P
Taxon
BRE
CFE

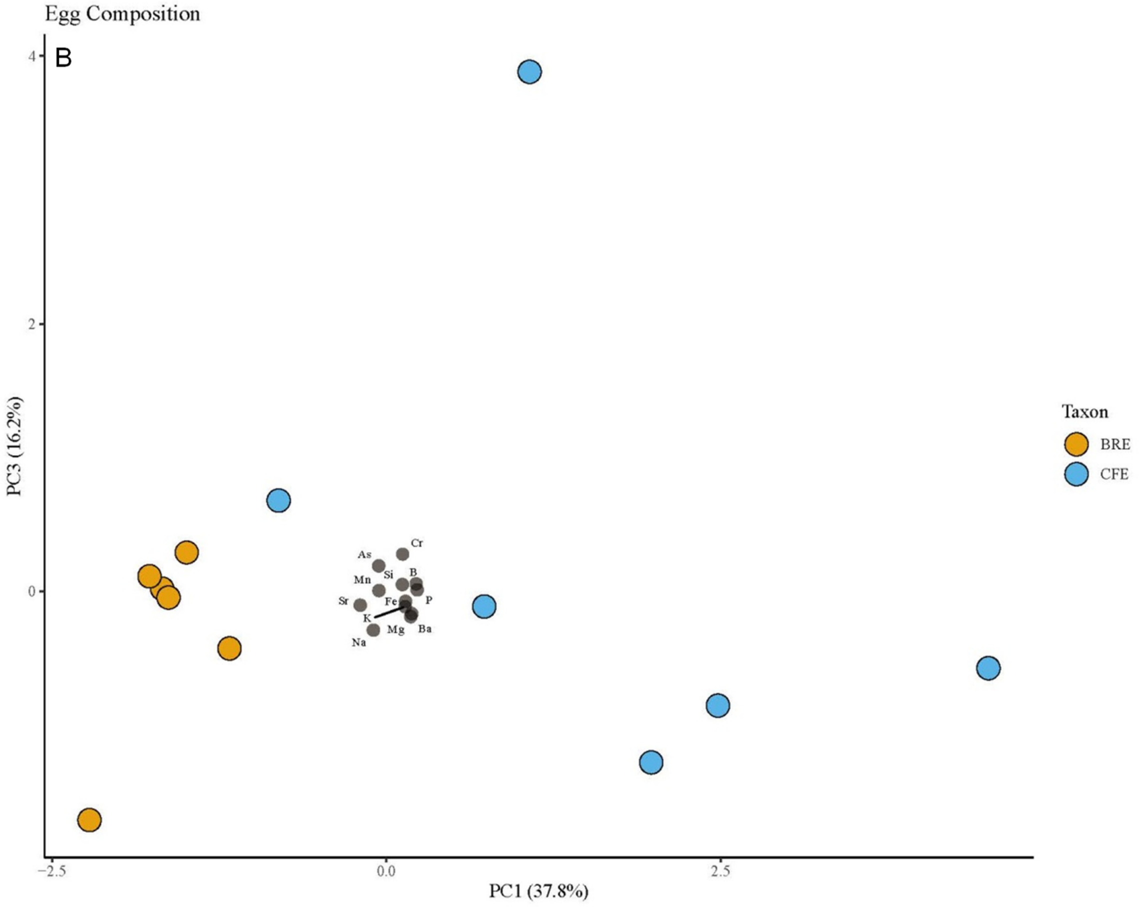

Egg Composition
B
PC3 (16.2%)
PC1 (37.8%)
4
2
0
−2.5
0.0
2.5
Cr
As
B
Si
Mn
Sr
Fe
P
K
Mg
Ba
Na
Taxon
BRE
CFE

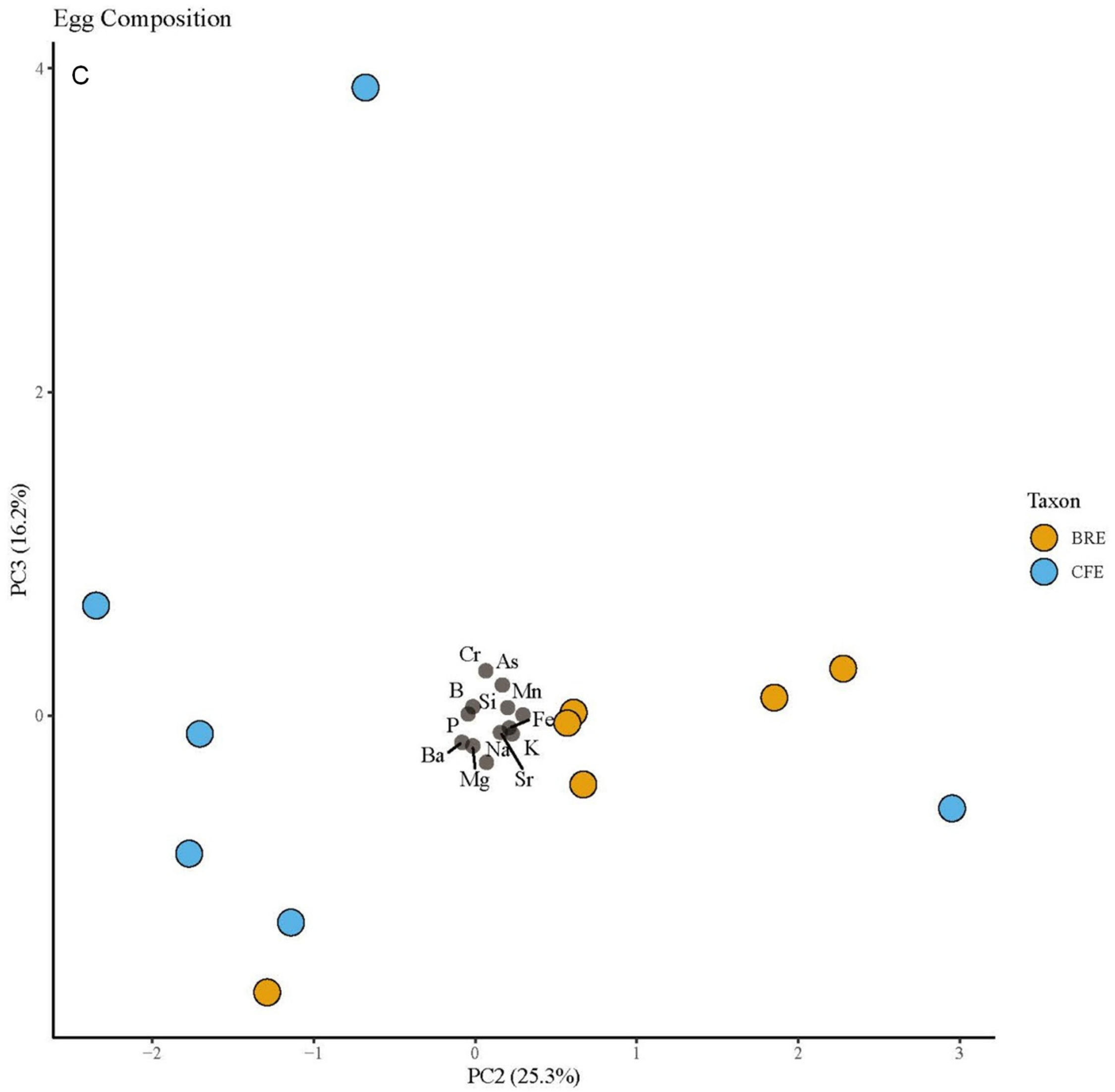

Figure S2. Results of principal component analysis (PCA) of Si, Na, Mg, K, Fe, Sr, P, Ba, B, Cr, As, and Mn in the eggs of the land snails *Bradybaena ravida* and *Cathaica fasciola*. Graphs A, B, and C show that the elemental compositions of the two egg groups differ greatly, demonstrating clear interspecific differentiation. BRE: *Bradybaena ravida* eggshells. CFE: *Cathaica fasciola* eggshells.

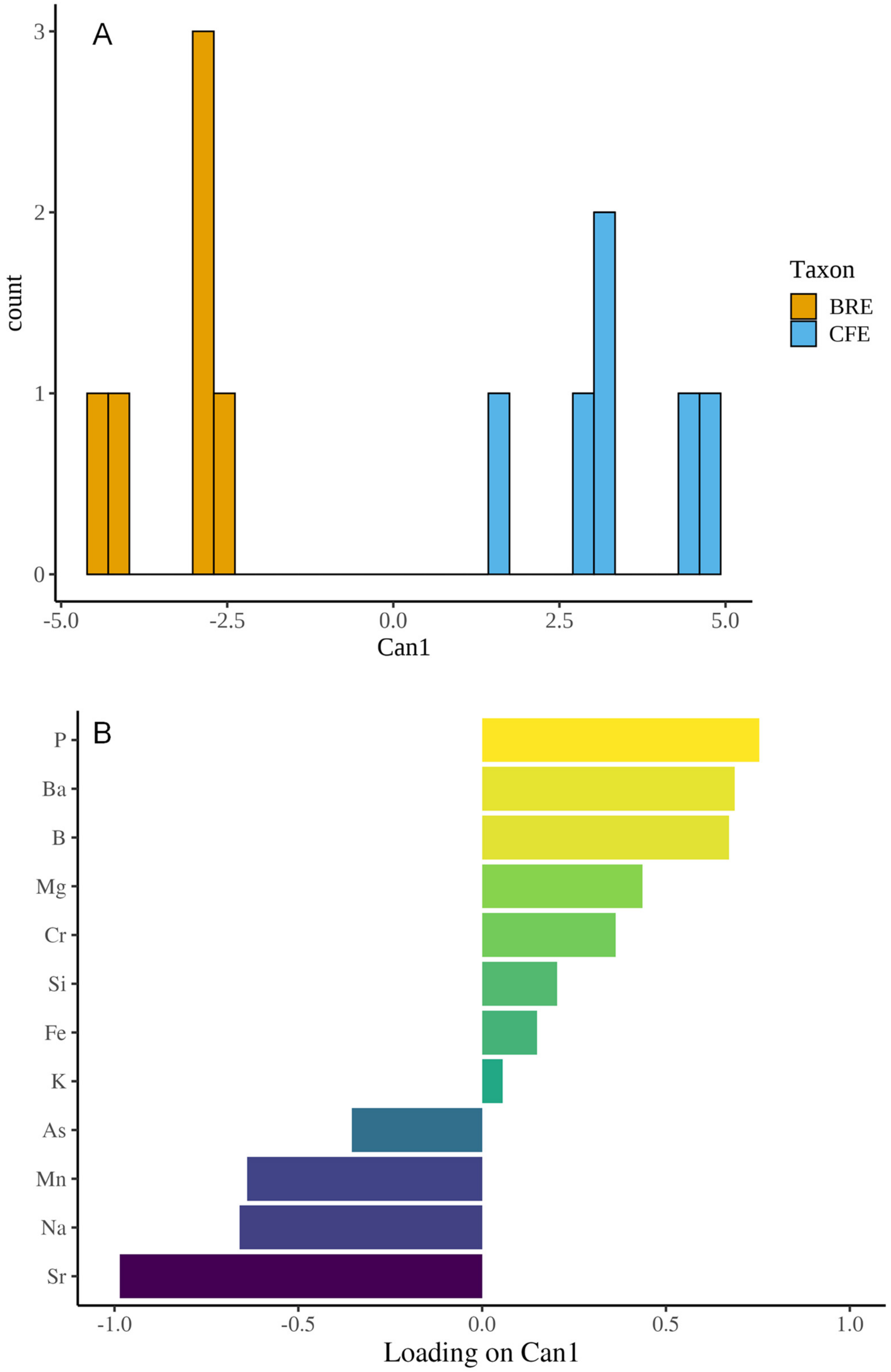

Figure S3. Results of canonical variate analysis (CVA) of Si, Na, Mg, K, Fe, Sr, P, Ba, B, Cr, As, and Mn in the eggshells of the land snails *Bradybaena ravida* and *Cathaica fasciola*. BRE: *Bradybaena ravida* eggshells. CFE: *Cathaica fasciola* eggshells.

Table S1. Mean, standard deviation (SD), and median of elemental concentrations in *Bradybaena ravida* and *Cathaica fasciola* and their eggshells.

Element	*Bradybaena ravida*			The eggshells of *Bradybaena ravida*			*Cathaica fasciola*			The eggshells of *Cathaica fasciola*		
	Mean (µg/g)	SD (µg/g)	Median (µg/g)	Mean (µg/g)	SD (µg/g)	Median (µg/g)	Mean (µg/g)	SD (µg/g)	Median (µg/g)	Mean (µg/g)	SD (µg/g)	Median (µg/g)
Si	638	286	655	1256	674	1178	916	566	770	1511	665	1302
Na	287	157	279	1518	164	1535	34.4	26.6	29.5	1198	230	1262
Mg	59.7	22.8	59.1	862	103	869	55.5	19.4	54.8	1085	341	1076
K	507	286	507	99.7	38.2	99.7	698	287	702	110	138	55.9
Fe	61.7	11.8	61.0	112	40.3	98.1	44.5	11.3	43.0	161	248	57.9
Sr	207	11.3	207	479	30.4	485	419	31.6	426	224	16.4	225
P	15.3	7.77	13.4	116	30.6	118	10.9	3.0	10.0	273	102	263
Ba	19.3	3.78	19.9	54.5	14.4	53.2	14.2	1.78	13.9	97.2	31.9	112.3
B	27.2	12.2	26.5	24.0	2.87	24.3	13.2	1.50	13.2	31.8	5.96	33.8
Cr	3.14	1.57	3.13	4.85	3.32	4.62	3.25	2.66	3.64	8.24	5.88	5.61
As	7.88	4.10	6.53	8.54	4.65	7.38	7.28	2.72	6.63	5.81	3.11	5.20
Mn	1.67	0.82	1.96	7.69	2.95	8.00	4.92	3.57	4.49	3.20	2.97	2.44
Al	1.81	2.18	1.08	94.0	65.6	94.5	0.008	0.021	0.000	308	424	80.3
Ti	0.411	0.891	0.000	19.8	26.6	9.28	0.458	0.732	0.000	51.6	103	5.04
Zn	38.4	92.2	0.157	2.02	1.30	2.52	0.103	0.134	0.037	1.11	1.28	0.789
Li	15.0	36.7	0.000	0.000	0.000	0.000	0.033	0.081	0.000	0.685	0.853	0.319
Cu	0.150	0.368	0.000	1.94	1.43	2.08	0.228	0.220	0.188	1.17	0.919	1.14
Ni	0.769	1.25	0.249	0.832	0.992	0.411	0.041	0.101	0.000	0.383	0.594	0.000
Sn	0.035	0.054	0.000	0.174	0.220	0.091	0.041	0.101	0.000	0.394	0.584	0.146
Pb	0.029	0.021	0.031	0.490	0.348	0.387	0.010	0.018	0.002	0.601	0.346	0.559
La	0.024	0.007	0.025	0.049	0.014	0.051	0.030	0.012	0.032	0.235	0.457	0.056
U	0.000	0.001	0.000	0.028	0.044	0.010	0.029	0.068	0.001	0.228	0.444	0.041
V	0.023	0.022	0.013	0.477	0.352	0.349	0.007	0.016	0.000	0.383	0.319	0.263
Rb	0.060	0.041	0.057	0.231	0.240	0.146	0.027	0.067	0.000	0.211	0.419	0.051
Nd	0.008	0.011	0.003	0.032	0.023	0.035	0.014	0.013	0.013	0.190	0.412	0.024
Y	0.005	0.007	0.002	0.037	0.030	0.037	0.008	0.007	0.010	0.208	0.401	0.054
Ce	0.012	0.007	0.011	0.140	0.134	0.096	0.030	0.013	0.025	0.177	0.323	0.057
Cd	0.042	0.071	0.000	0.366	0.357	0.347	0.040	0.064	0.000	0.026	0.041	0.000
Ag	0.014	0.024	0.003	0.024	0.036	0.014	0.006	0.010	0.000	0.130	0.302	0.005
Zr	0.034	0.049	0.005	0.155	0.225	0.071	0.076	0.095	0.035	0.119	0.237	0.020
Be	0.037	0.081	0.000	0.108	0.142	0.050	0.099	0.191	0.000	0.097	0.112	0.078
Ge	0.059	0.096	0.000	0.208	0.159	0.243	0.074	0.083	0.052	0.082	0.078	0.079
Ga	0.051	0.042	0.043	0.021	0.018	0.024	0.008	0.013	0.000	0.074	0.104	0.039
Gd	0.008	0.006	0.010	0.023	0.032	0.014	0.008	0.014	0.003	0.052	0.110	0.006
Pr	0.002	0.002	0.002	0.012	0.011	0.011	0.001	0.001	0.000	0.050	0.103	0.010
Co	0.033	0.020	0.035	0.107	0.086	0.103	0.048	0.050	0.042	0.075	0.096	0.040
Sb	0.012	0.020	0.000	0.036	0.034	0.032	0.018	0.025	0.011	0.061	0.090	0.029
Sm	0.000	0.000	0.000	0.003	0.007	0.000	0.010	0.016	0.000	0.043	0.095	0.000
Dy	0.002	0.004	0.000	0.020	0.018	0.024	0.003	0.005	0.000	0.038	0.065	0.013
Sc	0.013	0.023	0.001	0.061	0.043	0.060	0.023	0.036	0.000	0.053	0.075	0.011
Nb	0.001	0.001	0.000	0.027	0.050	0.008	0.002	0.002	0.002	0.037	0.043	0.030
Th	0.000	0.000	0.000	0.015	0.012	0.018	0.000	0.000	0.000	0.030	0.048	0.011
Cs	0.018	0.017	0.018	0.006	0.010	0.001	0.008	0.020	0.000	0.031	0.049	0.000
Er	0.005	0.007	0.003	0.004	0.010	0.000	0.005	0.005	0.006	0.020	0.038	0.005
Eu	0.002	0.004	0.000	0.002	0.005	0.000	0.002	0.004	0.000	0.014	0.030	0.000
Bi	0.002	0.003	0.000	0.005	0.006	0.003	0.013	0.028	0.001	0.007	0.009	0.004
In	0.006	0.007	0.003	0.003	0.006	0.000	0.000	0.000	0.000	0.017	0.017	0.013
Ho	0.000	0.001	0.000	0.001	0.002	0.000	0.001	0.002	0.000	0.015	0.017	0.010
Tb	0.001	0.001	0.000	0.002	0.003	0.002	0.001	0.002	0.001	0.007	0.013	0.002
Hf	0.002	0.003	0.000	0.005	0.013	0.000	0.002	0.005	0.000	0.000	0.000	0.000
Yb	0.006	0.005	0.007	0.000	0.000	0.000	0.008	0.009	0.008	0.000	0.000	0.000
Ta	0.000	0.000	0.000	0.006	0.005	0.006	0.001	0.004	0.000	0.004	0.008	0.000
Tm	0.000	0.001	0.000	0.003	0.004	0.002	0.000	0.001	0.000	0.002	0.004	0.000
Lu	0.001	0.002	0.000	0.002	0.002	0.000	0.001	0.001	0.000	0.001	0.001	0.000

Table S2. *T*-test results of five elements from land-snail eggshells that can potentially differentiate eggs of *Bradybaena ravida* and *Cathaica fasciola*.

Element	Eggshells of *Bradybaena ravida* (Mean ± SD, µg/g)	Eggshells of *Cathaica fasciola* (Mean ± SD, µg/g)	$t_{(10)}$	p	Significance
Ba	54.5 ± 14.4	97.2 ± 31.9	-2.99	0.014	*
Mg	862 ± 103	1084 ± 341	-1.53	0.157	ns
Na	1518 ± 164	1198 ± 230	2.78	0.02	*
P	116 ± 31	273 ± 101	-3.63	0.005	**
Sr	479 ± 30	224 ± 17	18.04	< 0.001	***

Note: $t_{(10)}$ represents the *t*-statistic with 10 degrees of freedom.
p value: probability value of an independent-sample t-test.
Significance levels: *: $p < 0.05$, **: $p < 0.01$, ***: $p < 0.001$; ns: no significant difference ($p > 0.05$).

R code

```
library(readxl)
library(tidyverse)
library(ggrepel)
library(ggplot2)

EggPCAdata <- read_excel(file.choose(),sheet = 2)

EggPCAdata <- as.data.frame(EggPCAdata)
rownames(EggPCAdata) <- EggPCAdata[, 1]

EggPCAdata <- EggPCAdata[, -1]

okabe_ito_colors <- c("#E69F00", "#56B4E9", "#009E73", "#F0E442", "#0072B2", "#D55E00",
"#CC79A7", "#999999", "#990000", "#660066", "#00CC99")
EggPCA <- prcomp(EggPCAdata[,c(1:12)], center = TRUE, scale. = TRUE)
summary(EggPCA)


EggPCAdata <- EggPCAdata %>%
  bind_cols(EggPCA$x)
```

```
# Extract EggPCA loadings (variable contributions)
eggpca_loadings <- as.data.frame(EggPCA$rotation)    # Loadings for each element
eggpca_loadings$Element <- rownames(eggpca_loadings)    # Keep element names


egg_scaling_factor <- max(abs(c(eggpca_loadings$PC1, eggpca_loadings$PC2,
eggpca_loadings$PC3)))

# Scale the loadings for each component (PC1, PC2, and PC3)
eggpca_loadings_scaled <- eggpca_loadings
eggpca_loadings_scaled[, c("PC1", "PC2", "PC3")] <- eggpca_loadings[, c("PC1", "PC2", "PC3")] *
egg_scaling_factor

#Plot EggPCA results
#PC1 v PC2
p1 <- ggplot() +
   geom_text_repel(data = eggpca_loadings_scaled, aes(PC1, PC2, label = Element),family =
'serif') +
  geom_point(data = eggpca_loadings_scaled, aes(PC1, PC2), alpha=0.5,size=3) +
   geom_point(data = EggPCAdata, aes(PC1, PC2,fill = Taxon), shape=21, size=6)+
xlab("PC1 (37.8%)") + ylab("PC2 (25.3%)") + ggtitle("Egg Composition")+
   coord_fixed() + scale_fill_manual(values = okabe_ito_colors) + theme_classic() +
   theme(text = element_text(family = 'serif'))
p1
dev.off()


#PC2 v PC3

p2 <- ggplot() + geom_text_repel(data = eggpca_loadings_scaled, aes(PC2, PC3, label =
Element),family = 'serif') +
   geom_point(data = eggpca_loadings_scaled, aes(PC2, PC3), alpha=0.5, size=3) +
      geom_point(data = EggPCAdata, aes(PC2, PC3, fill = Taxon), shape=21, size=6)    +
      xlab("PC2 (25.3%)") + ylab("PC3 (16.2%)") + ggtitle("Egg Composition") +
     coord_fixed() + scale_fill_manual(values = okabe_ito_colors) + theme_classic() +
      theme(text = element_text(family = 'serif'))
p2


#PC1 v PC3

P3 <- ggplot() + geom_text_repel(data = eggpca_loadings_scaled, aes(PC1, PC3, label =
Element),family = 'serif',size =2.5) +
   geom_point(data = eggpca_loadings_scaled, aes(PC1, PC3), alpha=0.5, size=3) +
```

```
  geom_point(data = EggPCAdata, aes(PC1, PC3, fill = Taxon), shape=21, size=6)    +
  xlab("PC1 (37.8%)") + ylab("PC3 (16.2%)") + ggtitle("Egg Composition") +
  coord_fixed() + scale_fill_manual(values = okabe_ito_colors) + theme_classic() + theme(text =
element_text(family = 'serif'))
P3


#### nmds

library(vegan)
set.seed(123)

EggPCAdata <- read_excel(file.choose(),sheet = 2)

EggPCAdata <- as.data.frame(EggPCAdata)
rownames(EggPCAdata) <- EggPCAdata[, 1]

EggPCAdata <- EggPCAdata[, -1]
EggNMDSdata <- EggPCAdata
EggNMDS <- metaMDS(EggNMDSdata[,c(1:12)], distance = "bray", k=2, trymax = 100)

Eggspecimen_scores <- as.data.frame(EggNMDS$points)
EggNMDSscores <- EggNMDSdata %>%
  bind_cols(Eggspecimen_scores)
EggNMDSvariablescores <- as.data.frame(EggNMDS$species)
EggNMDSvariablescores$Element <- rownames(EggNMDSvariablescores)


p5 <- ggplot() + geom_text_repel(data = EggNMDSvariablescores, aes(MDS1, MDS2, label =
Element),family = 'serif',size = 4) +
  geom_point(data = EggNMDSvariablescores, aes(MDS1, MDS2), alpha=0.5,size = 3) +
  geom_point(data = EggNMDSscores, aes(MDS1, MDS2, fill=Taxon), shape=21, size=7) +
  ggtitle("Egg Composition") + annotate(geom = "text", x=-0.15, y=-0.15, label="Stress=0.07") +
  coord_fixed() + scale_fill_manual(values = okabe_ito_colors) + theme_classic() +
  theme(text = element_text(family = 'serif'))

p5


####CVA

library(candisc)

EggPCAdata <- read_excel(file.choose(),sheet = 2)
```

```
EggPCAdata <- as.data.frame(EggPCAdata)
rownames(EggPCAdata) <- EggPCAdata[, 1]

EggPCAdata <- EggPCAdata[, -1]

EggPCAdata$Taxon <- as.factor(EggPCAdata$Taxon)

EggCVAdata <- EggPCAdata
manova_result <- manova(as.matrix(EggCVAdata[, c(1:12)]) ~ Taxon, data = EggCVAdata)
#Conduct CVA on Manova
cva_result <- candisc(manova_result, term = "Taxon")
#Extract CVA scores
cva_scores <- as.data.frame(cva_result$scores)
cva_scores$Texon <- EggPCAdata$Texon    # Add group labels

cva_loadings <- as.data.frame(cva_result$structure)
cva_loadings$Element <- rownames(cva_loadings)

#Plot CVA results
p7 <- ggplot(data = cva_scores) + geom_histogram(aes(Can1, fill=Taxon), color="black") +
  scale_fill_manual(values = okabe_ito_colors) + theme_classic() + theme(text =
element_text(family = 'serif'))
p7


p8 <- ggplot(cva_loadings, aes(x = reorder(Element, Can1), y = Can1, fill = Can1)) +
  geom_bar(stat = "identity", show.legend = FALSE) +
  coord_flip() + scale_fill_viridis_c() +
  theme_classic() + xlab("") +
  ylab("Loading on Can1") + ylim(c(-1,1)) + theme(text = element_text(family = 'serif'))
p8
```
